# Molecular Epidemiology of *Mycobacterium tuberculosis*, Buenos Aires, Argentina

**DOI:** 10.3201/eid1703100394

**Published:** 2011-03

**Authors:** Ximena Gonzalo, Marta Ambroggi, Ezequiel Cordova, Tim Brown, Susana Poggi, Francis Drobniewski

**Affiliations:** Author affiliations: Hospital de Infecciosas Francisco Javier Muñiz, Buenos Aires, Argentina (X. Gonzalo, M. Ambroggi, E. Cordova, S. Poggi);; Clinical Sciences Research Center, London, UK (T. Brown, F. Drobniewski)

**Keywords:** Tuberculosis, tuberculosis and other mycobacteria, molecular epidemiology, drug resistance, Argentina, Mycobacterium tuberculosis, dispatch

## Abstract

To analyze the molecular epidemiology of *Mycobacterium tuberculosis* strains at a hospital in Buenos Aires, Argentina, and mutations related to multidrug-resistant and extensively drug-resistant tuberculosis, we conducted a prospective case–control study. Our findings reinforce the value of incorporating already standardized molecular methods for rapidly detecting resistance.

During the 1990s, an outbreak of multidrug-resistant (MDR) tuberculosis (TB) in HIV-positive patients occurred at the Muñiz Hospital in Buenos Aires, Argentina (*1*). Molecular analysis showed that a member of Haarlem2 family of *Mycobacterium tuberculosis* was responsible (*1*).

We conducted a prospective case-control study during June 1, 2006–April 30, 2007, at this 300-bed public hospital, which reports ≈40% of new TB cases in the city (*2*). Our primary aims were to analyze the molecular epidemiology of *M. tuberculosis* strains circulating at the hospital and the mutations related to MDR TB and extensively drug-resistant TB.

## The Study

The strains were isolated and tested for antimicrobial drug susceptibility according to the proportion method (*3*) at the Muñiz Hospital Mycobacteria Laboratory. A proportion were tested for reserve drugs at the Health Protection Agency National Mycobacteria Reference Unit Laboratory, London, UK. DNA was extracted from cultures at this UK laboratory; spoligotyping was performed according to the manufacturer’s instructions (Isogen Life Science, IJsselstein, the Netherlands); and data were analyzed with SPOTCLUST (http://cgi2.cs.rpi.edu/~bennek/SPOTCLUST.html).

We performed 15-locus variable number tandem repeat (VNTR) using a CEQ 8000 Genetic Analysis System (Beckman Coulter, Inc., Fullerton, CA, USA) (*4*). Cluster analysis was performed by using Bionumerics software (Applied Maths, St-Martens-Latem, Belgium). Strains lacking a unique pattern were subjected to further analysis with an expanded set of VNTR loci (*5*).

In-house macroarrays were performed on MDR TB strains (*6*) to identify mutations in the *katG* and the *inhA* genes. Two regions of the *rpoB* gene of MDR TB strains were sequenced with the CEQ 8000 Genetic Analysis System. Statistical analyses were conducted by using χ^2^ and Fisher exact tests.

**Figure 1:**
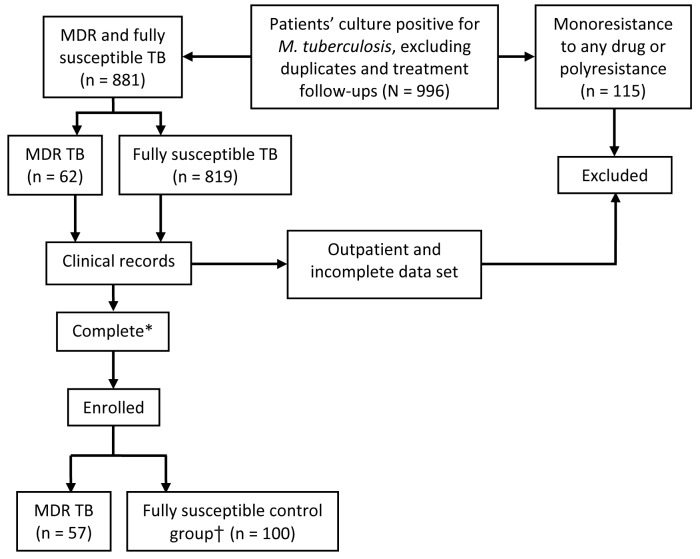
Multistage cluster sampling method for a study of the molecular epidemiology of *Mycobacterium tuberculosis*, Buenos Aires, Argentina, June 1, 2006–April 30, 2007. *Complete, having all but one of the following data: name, gender, date of birth or age, tuberculosis (TB) presentation, >1 sign or symptom describing the presentation and treatment received. †Unmatched controls. MDR, multidrug-resistant.

After we excluded duplicates, treatment follow-ups, and strains with susceptibility patterns other than MDR TB or susceptibility to all drugs tested, 881 strains were susceptible to all drugs tested. Patients with a minimum dataset (name, sex, date of birth or age, TB presentation [i.e., pulmonary or nonpulmonary], and at least 1 sign or symptom describing TB illness and treatment received) were enrolled: 57 of 62 hospitalized patients with MDR TB cultures (Table) and 100 fully susceptible unmatched inpatient controls, for a total of 157 patients. This convenience sample (Figure 1) included only admitted patients because of the difficulty of obtaining clinical information about outpatients.

**Table:** Demographic information for 157 patients in a study of the molecular epidemiology of TB, Buenos Aires, Argentina, June 1, 2006–April 30, 2007*

Demographic characteristic	Patients with MDR TB, n = 57, no. (%)†	Patients with non–MDR TB, n = 100, no. (%)‡	p value, OR (95% CI)§
Sex			
M	35 (61)	70 (70)	0.271, 1.4667 (0.7404–2.9055)
F	22 (39)	30 (30)	
Location			
Buenos Aires area	46 (81)	95 (95)	0.004, 4.5435 (1.491–13.845)
Other	11 (19)	5 (5)	
Country of birth			
Argentina	43 (75)	66 (66)	0.2176, 0.632 (0.3041–1.3133)
Bolivia	6 (11)	20 (20)	
Peru	7 (12)	8 (8)	
Paraguay	0	3 (3)	
Uruguay	1 (2)	1 (1)	
Chile	0	1 (1)	
Missing data	0	1 (1)	
Education			
Illiterate or some primary	16 (28)	32 (32)	0.2059, 0.5185 (0.1860–1.4456)
Some secondary or tertiary	7 (12)	27 (27)	
Missing data	34 (59)	41 (41)	
Occupation			
Unemployed	7 (12)		
Construction and manual worker	20 (35)		
Factory worker	4 (7)	14 (14)	
Health care worker	1 (2)	1 (1)	
Education, i.e., student and teacher	2 (4)	4 (4)	
Housewife	6 (11)	5 (5)	
Missing data	17 (30)	23 (23)	
HIV infection			
Positive	25 (44)	27 (27)	0.04, 0.4737 (0.2308–0.9722)
Negative	25 (44)	57 (57)	
Missing data	7 (12)	16 (16)	
Nature of TB contact			
Close (i.e., household, family, co-worker)	10 (18)	32 (32)	
Institution (i.e., hospital, prison)	2 (4)	3 (3)	
Casual (e.g., acquaintance)	5 (9)	3 (3)	
Missing data	40 (70)	62 (62)	
TB presentation			
Pulmonary	36 (63)	61 (61)	0.7184, 1.1553 (0.5323–2.5073)
Nonpulmonary	15 (26)	22 (22)	
Missing data	6 (11)	17 (17)	
*TB, tuberculosis; MDR, multidrug resistant; OR, odds ratio; CI, confidence interval; IQR, interquartile range. †Median age, y (IQR) for patients with MDR TB: 34 (27–40). ‡Median age, y (IQR) for patients with non-MDR TB: 28.5 (23.0–37.0). §χ^2^ test.			

**Figure 2:**
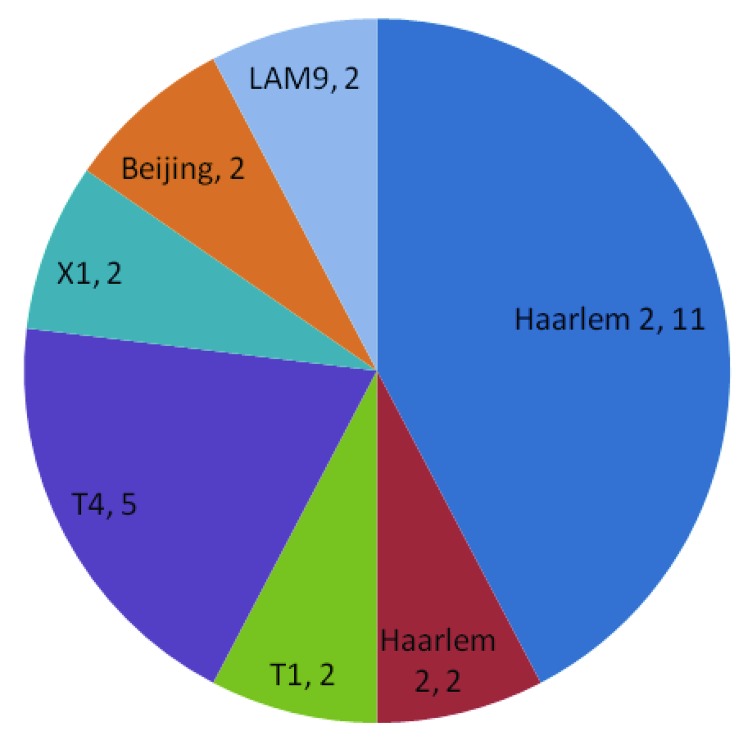
Final cluster results in a study of the molecular epidemiology of *Mycobacterium tuberculosis*, by spoligofamily, Buenos Aires, Argentina, June 1, 2006–April 30, 2007.

The most common spoligofamilies were T1 (18%), Haarlem 1 (10%), 2 (16%), and 3 (11%), LAM 3 (10%), and LAM 9 (13%). Initial 15-loci VNTR analysis showed 73 strains with unique patterns. Further analysis using 7-loci VNTR was performed on those that did not have a unique pattern. Twenty-six isolates remained clustered (Figure 2).

Of the 57 MDR TB strains, 43 had a mutation in the *katG315* locus, and 6 had a mutation in the *inhA* region. No strain had mutations in both genes. In 8 strains, resistance to isoniazid was not mediated by mutations in any of them.

Mutations in the *rpoB* region were detected by sequencing. The most frequent mutation was the S531L. Mutations at >1 site were rare. In 3 cases, we found 2 point mutations in the same codon. Only 1 MDR TB strain had no mutation in the *rpo*B segment sequenced; it also had a *kat*G and *inh*A wild type. Complete susceptibility profile for all 57 MDR TB strains is available in Table A1.

In addition, we detected 5 extensively drug-resistant TB strains. None were clustered by 22-loci VNTR typing.

## Conclusions

Spoligotyping identified predominance of the Haarlem family among the MDR TB cases (family responsible for the 1990s [*1*] outbreak) as well as the LAM and T families. A similar strain family distribution was reported for the French Departments of the Americas (*7*) and Turkey (*8*). The Beijing family was seldom encountered in these areas, which is in line with recent observations in 7 countries in South America, including Argentina (*9*).

The MDR TB Haarlem2 strain appears to be more successful than other circulating MDR TB strains and than its susceptible counterpart (of 25 Haarlem2 strains, 20 were MDR TB). This phenomenon could be associated with a bias in the sample resulting from the specialized nature of the hospital or it could be that the MDR TB version has become predominant in the population because of the low fitness cost of its 2 mutations, *kat*G315 and S531L (*10*,*11**)*)). In addition, the presence of clusters suggests that even though new technologies have reduced the time taken to diagnose drug resistance, more rapid initial diagnosis of MDR TB to reduce transmission still is needed (*12*).

All except 1 of the *rpo*B mutations in the MDR TB strains were at nt positions 1303–1375. This finding reinforces the value of incorporating already standardized molecular methods for rapidly detecting resistance. Cost is the main reason they are currently not available, but the macroarrays used in this project are inexpensive and have the additional advantage of analyzing *kat*G and *inh*A mutations independently.

## Appendix

**Table A1:** Drug susceptibility profiles for 57 patients with MDR TB, Buenos Aires, Argentina, June 1, 2006–April 30, 2007*

Patient no.	INH	Rif	P	E	Str	PAS	Cs	Pto	Eto	Cm	Am	Km	Cip	Ofx	Lfx	Mfx
1	R	R	S	S	R	S	S	–	–	–	–	S	–	–	–	–
2	R	R	R	–	R	–	–	–	S	–	–	–	–	–	–	–
3	R	R	S	S	R	S	–	–	–	–	–	S	–	–	–	–
4	R	R	R	R	R	S	S	–	–	–	–	R	–	R	–	–
5	R	R	R	R	R	S	S	–	–	–	–	–	–	–	–	–
6	R	R	R	R	R	R	S	–	–	S	–	R	–	S	–	S
7	R	R	–	R	R	–	–	–	–	–	–	–	–	–	–	–
8	R	R	R	S	R	S	S	–	–	–	–	R	–	–	–	–
9	R	R	–	S	R	S	S	–	–	–	–	–	–	–	–	–
10	R	R	R	R	R	–	–	–	–	–	R	–	–	–	–	–
11	R	R	R	R	R	S	S	–	–	–	–	–	–	–	–	–
12	R	R	R	R	R	S	S	–	–	–	–	–	–	–	–	–
13	R	R	R	R	R	S	S	–	–	–	–	–	–	–	–	–
14	R	R	S	R	R	S	S	–	–	–	–	–	–	–	–	–
15	R	R	R	R	R	S	S	S	S	R	R	R	–	S	–	S
16	R	R	R	R	R	–	–	S	S	R	R	R	–	S	–	S
17	R	R	R	R	R	S	S	–	–	–	–	R	–	–	–	–
18	R	R	R	R	R	S	S	S	R	R	R	R	–	S	–	S
19	R	R	R	R	R	S	S	S	S	R	R	R	S	S	–	S
20	R	R	R	S	R	S	–	S	S	R	R	R	–	S	–	S
21	R	R	R	R	R	–	–	S	S	R	R	R	S	S	–	S
22	R	R	R	R	S	S	–	–	–	–	–	R	–	–	–	–
23	R	R	R	R	R	–	–	–	–	R	R	R	–	R	–	R
24	R	R	R	R	R	S	R	R	R	S	S	R	–	S	–	S
25	R	R	S	R	R	S	S	–	–	–	–	S	–	–	–	–
26	R	R	R	R	R	S	S	–	–	–	–	–	–	–	–	–
27	R	R	R	R	R	R	S	–	–	–	–	R	–	R	–	–
28	R	R	R	R	R	R	S	–	–	–	–	R	–	S	–	–
29	R	R	R	–	S	S	S	–	–	–	–	–	–	–	–	–
30	R	R	R	S	S	S	S	–	–	–	–	S	–	–	–	–
31	R	R	S	S	R	S	S	–	–	–	–	S	–	–	–	–
32	R	R	S	S	R	S	S	–	–	–	–	S	–	–	–	–
33	R	R	R	R	S	S	–	–	–	–	–	S	–	–	–	–
34	R	R	–	S	R	S	–	–	–	–	–	–	–	–	–	–
35	R	R	R	R	S	S	S	–	–	–	–	S	–	S	–	–
36	R	R	S	R	S	S	S	–	–	–	–	–	–	–	–	–
37	R	R	S	S	S	S	–	–	–	–	–	–	–	–	–	–
38	R	R	R	S	S	S	S	–	–	–	–	S	–	–	–	–
39	R	R	R	R	R	S	S	–	–	–	R	R	–	R	–	–
40	R	R	S	R	R	S	S	–	–	S	–	S	–	S	–	–
41	R	R	R	R	S	S	S	–	–	–	–	S	–	–	–	–
42	R	R	–	R	R	S	S	–	–	–	–	R	–	–	–	–
43	R	R	–	S	S	S	–	S	R	S	S	S	–	S	–	S
44	R	R	R	R	R	R	S	–	–	–	–	–	–	S	–	–
45	R	R	R	R	R	S	S	–	–	–	–	R	–	–	–	–
46	R	R	R	R	R	S	S	–	–	–	–	R	–	–	–	–
47	R	R	S	S	R	S	–	–	–	–	–	S	–	–	–	–
48	R	R	R	S	S	S	–	–	–	–	–	–	–	–	–	–
49	R	R	S	S	R	S	S	–	–	–	–	S	–	–	–	–
50	R	R	R	R	R	R	R	–	–	S	–	R	R	R	–	S
51	R	R	S	R	S	S	–	–	–	–	–	–	–	–	–	–
52	R	R	S	S	S	S	S	–	–	–	–	–	–	–	–	–
53	R	R	R	S	S	S	–	R	R	–	–	S	–	S	–	–
54	R	R	R	R	R	S	S	–	–	–	–	R	–	–	–	–
55	R	R	–	S	S	–	–	–	–	–	–	–	–	–	–	–
56	R	R	–	S	S	S	S	–	–	–	–	S	–	–	–	–
57	R	R	S	R	R	S	S	–	–	–	–	S	–	–	–	–

*MDR, multidrug resistant; TB, tuberculosis; INH, isoniazid; Rif, rifampicin; P, pyrazinamide; E, ethambutol; Str, streptomycin; PAS, p-aminosalicylic acid; Cs, cycloserine; Pto, proteonamide; Eto, ethionamide; Cm, capreomycin; Am, amikacin; Km, kanamycin; Cip, ciprofloxacin; Ofx, ofloxacin; Lfx, levofloxacin; Mfx, moxifloxacin; R, resistant; S, susceptible; –, drugs not tested for those strains.
